# The versatility of 18ß-glycyrrhetinic acid in attenuating pulmonary inflammatory disorders

**DOI:** 10.17179/excli2023-5845

**Published:** 2023-02-06

**Authors:** Mohamad Siddiq Bin Mohamad, Ruby-Jean Reyes, Gabriele De Rubis, Keshav Raj Paudel, Philip Michael Hansbro, Kamal Dua, Dinesh Kumar Chellappan

**Affiliations:** 1 School of Postgraduate Studies, International Medical University, Bukit Jalil 57000, Kuala Lumpur, Malaysia; 2 Faculty of Health, Australian Research Center in Complementary & Integrative Medicine, University of Technology Sydney, 2007, Ultimo, Australia; 3 Discipline of Pharmacy, Graduate School of Health, University of Technology Sydney, Sydney, NSW 2007, Australia; 4 Center of Inflammation, Centenary Institute and University of Technology Sydney, Faculty of Science, School of Life Sciences, Sydney 2007, Australia; 5 Uttaranchal Institute of Pharmaceutical Sciences, Uttaranchal University, Dehradun 248007, India; 6 Department of Life Sciences, School of Pharmacy, International Medical University, Bukit Jalil 57000, Kuala Lumpur, Malaysia

## ⁯

Pulmonary inflammatory disorders encompass a diverse spectrum of diseases including asthma, chronic obstructive pulmonary diseases, lung fibrosis, lung infection, and lung cancer. Inflammation is a physiological response occurring in the human body whenever there is a threat in the form of an infection as well as a non-infectious, harmful damage, activating a myriad of cellular mechanisms that eventually ends with tissue repair. Inflammation in the lung normally occurs as a response to threats such as allergens, infective agents, irritants and, sometimes, tumors. However, excessive inflammation may progress past the tissue repair stage, further leading to deleterious effects, and advancing to pulmonary inflammatory disorders (Moldoveanu et al., 2009[10]). 

An estimated 300 million people worldwide suffer from asthma. The World Health Organization (WHO) has estimated that 250,000 asthma fatalities are reported globally each year, along with a loss of 15 million disability-adjusted life-years (Bateman et al., 2008[1]). Lung cancer is one of the most commonly diagnosed malignancies and the biggest cause of cancer-related deaths globally, with an estimated 2.2 million new cases and 1.79 million deaths each year (Ferlay et al., 2021[2]). This shows the alarming and scale of impact posed by pulmonary inflammatory disorders on human health globally, which needs to be addressed imminently. 

Fortunately, there are growing trends in applying naturally derived materials to produce medicine to treat pulmonary inflammatory disorders more efficiently. Among these naturally derived materials, 18β-glycyrrhetinic acid (18β-Gly) is gaining notable attention and importance. 18β-Gly is a pentacyclic triterpenoid metabolite hydrolyzed from glycyrrhizic acid of the licorice plant (Ming and Yin, 2013[9]). It is suitable for a wide range of potential clinical uses due to its broad spectrum of anti-oxidative, anti-inflammatory, and anti-neoplastic actions (Kowalska and Kalinowska-Lis, 2019[6]). Thus, 18β-Gly may offer promising medicinal treatment to combat life-threatening diseases such as pulmonary inflammatory disorders.

In recent years, numerous research articles have drawn attention to the potential pharmacological use of 18β-Gly. In the section below, we highlight the anti-inflammatory effects of 18β-Gly from *in vivo* studies. Liu et al. (2022[7]) conducted a study to evaluate the potential of 18β-Gly as an anti-inflammatory therapeutic using an ovalbumin (OVA)-induced asthma mouse model. The result showed that 18β-Gly significantly attenuated inflammation and enhanced lung function of these asthmatic mouse models. Moreover, 18β-Gly blocked the OVA-induced phosphorylation of nuclear factor kappa-light-chain-enhancer of activated B cells (NF-κB) in the lungs of mice. Finally, 18β-Gly also elevated the nuclear factor erythroid 2-related factor 2 (Nrf2) and heme oxygenase-1 (HO-1) expression. This study concluded that 18β-Gly posed as a viable treatment option for asthma (Liu et al., 2022[7]). In another study by Kim et al. (2017[5]), the mechanism of action of 18β-Gly in counteracting airway inflammation was investigated. The study indicated that 18β-Gly is able to inhibit RAR-related orphan receptor gamma (RORγt), signal transducer and activator of transcription 6 (STAT6), GATA binding protein 3 (GATA-3) pathways as well as upregulate forkhead box P3 (Foxp3) transcription, resulting in the suppression of interleukin (IL)-5, IL-13, and immunoglobin E production. 18β-Gly also protects against oxidative stress by suppressing MH-S alveolar macrophage cells ability to generate reactive oxygen species (ROS) (Kim et al., 2017[5]). These studies strengthen the potential of 18β-Gly as a viable novel anti-inflammatory agent. 

Furthermore, 18β-Gly also exhibits anti-neoplastic actions, confirmed by recent *in-vivo* research studies. Luo et al. (2021[8]) conducted a study to assess the anti-neoplastic effects of 18β-Gly on A549 lung cancer cells (Luo et al., 2021[8]). wherein this study, 18β-Gly showed a significant cytotoxic effect exerted through mitochondria-dependent apoptosis, G2/M cell cycle arrest, as well as migration inhibition through ROS/mitogen-activated protein kinases (MAPK)/signal transducer and activator of transcription 3 (STAT3)/NF-κB signaling pathways in A549 cells with no apparent side effects. This places 18β-Gly as a desired alternative option to treat lung cancer. Moreover, Huang et al. (2014[4]) discovered that 18β-Gly prevents non-small cell lung cancer cells proliferation through thromboxane synthase (TxAS) inhibition as well as extracellular signal-regulated kinase/cAMP responsive element binding protein (ERK/CREB) signaling initiation (Huang et al., 2014[4]). This indicates that 18β-Gly has potential for non-small cell lung cancer prevention and treatment. 

Despite the numerous research studies investigating the therapeutic potential of 18β-Gly, this molecule possesses unfavorable physicochemical characteristics such as poor bioavailability and low water solubility (Hao et al., 2010[3]). These characteristics prevents the application of 18β-Gly as a conventional treatment in clinical practice. In recent years, there has been a rapid progression of nanotechnology which, applied to the advanced delivery of phytoceuticals, is helping to improve the bioavailability of many of these molecules. Considering the great therapeutic potential shown by 18β-Gly, nanotechnology is widely acknowledged as a promising platform for researchers to formulate it into various types of nano-formulation that can improve bioavailability while maintaining the therapeutic potential of 18β-Gly. This in turn will help to create viable options to treat lung cancer and pulmonary inflammatory diseases more efficiently. The recent research studies highlighted above demonstrate the significant potential of 18β-Gly in treating pulmonary inflammatory disorders. The application of nanotechnology to create nano-based carriers for 18β-Gly will help to revolutionize treatment for pulmonary inflammatory disorders.

## Conflict of interest

The authors declare that they have no conflict of interest.
